# Supplementary material for: Complexome profiling on the Chlamydomonas lpa2 mutant reveals insights into PSII biogenesis and new PSII associated proteins
Source: J Exp Bot. 2021 Aug 26;73(1):245–62. doi: 10.1093/jxb/erab390 (PMC8730698; doi:10.1093/jxb/erab390)
Supplement: erab390_suppl_Supplementary_Dataset_S1 [file erab390_suppl_supplementary_dataset_s1.zip › Supplemental Dataset 1 - Excel List and all profiles/plots/CYP771A1_Cre08.g384000.html]

Trivial name: CYP771A1  
  
Euclidean distance: 39601.59  
Mean Intensity (WT): 3101.43  
Mean Intensity (Mut): 1699.37  
Distance: 12.77  
  
MapMan: misc.cytochrome P450;misc.misc2;hormone metabolism.brassinosteroid.synthesis-degradation.BRs.metabolic regulation  
  
p value of intensity sums Welch test: 0.1073
